# Supplementary material for: Are carbon emissions trading and green financial instruments synergistic? -Comprehensive quantitative research based on content analysis
Source: PLoS One. 2024 Mar 7;19(3):e0298601. doi: 10.1371/journal.pone.0298601 (PMC10919601; doi:10.1371/journal.pone.0298601)
Supplement: S1 Data — (ZIP) [file pone.0298601.s001.zip › Data/地级市Z_F工作报告.zip!/╔╜╢1⁄2/┴─│╟╩╨/└┤╫╘╣1⁄2╓┌║┼í╛┬φ┐╦╩2╛▌═°í┐_2004.docx]

各位代表：

现在，我代表市人民政府，向大会作政府工作报告，请予审议，并请市政协委员和其他列席会议的同志提出意见。

2003年政府工作的回顾

2003年是很不平凡的一年，是经济社会事业发展取得重大成效的一年，是抗击非典取得重大胜利的一年。一年来，全市人民高举邓小平理论和“三个代表”重要思想伟大旗帜，认真贯彻党的十六大、十六届三中全会和省、市委工作会议精神，在省委、省政府和中共聊城市委的坚强领导下，在历届市领导班子打下的良好基础上，解放思想，干事创业，团结实干，奋力拼搏，胜利完成了市十四届人大一次会议确定的各项任务，改革开放和现代化建设取得显著成就。据核算和统计，全市实现国内生产总值454.05亿元，比上年增长16.4%，其中一二三产业分别增长4.6%、24.1%和16.0%；地方财政收入20.24亿元，增长26.4%；金融机构存、贷款余额分别比年初增加72.5亿元和59.8亿元；城镇居民人均可支配收入6800元、农民人均纯收入2677元，分别增长9.5%和6.95%。经济发展速度明显加快，运行质量和效益明显提高，社会事业全面进步，人民生活继续改善，我市经济和社会发展进入了一个新的阶段！

一年来，我们重点抓了以下八个方面的工作：

（一）周密部署，筑牢防线，夺取了抗击非典斗争的重大胜利。去年上半年，面对突如其来的非典疫情，我们坚决贯彻落实党中央、国务院和省委、省政府的决策和部署，始终坚持把保护人民群众的身体健康和生命安全放在第一位，把防治非典作为重大任务来抓。在市委的统一领导下，各级政府精心组织，精心指挥，严密部署，严格责任，狠抓落实，层层建立了组织指挥、群防群治、隔离治疗、后勤保障四大体系，筑牢了交通检查、监测观察、门诊留验、隔离治疗四道防线。经过三个多月的艰苦奋战，全面实现了省委、省政府提出的“堵住源头不输入，一旦输入不扩散，及时救治不死亡，医护人员不感染”的目标，确保了无一例非典病例和疑似病例输入聊城，守住了山东的西大门。同时，坚持一手抓防治非典，一手抓经济发展，及时提出相应对策，努力减少非典造成的影响和损失，保持了经济发展的良好势头。

（二）坚定不移地抓招商、增投入，培植形成了一批新的经济增长点。把招商引资作为经济发展的总抓手，认真实施“大开放、大招商，促进大发展”战略。非典期间，主动请进来、走出去，抓紧工作不放松；疫情过后，迅速出击，在韩国、日本、香港、深圳、昆山等国家、地区和城市举办了一系列重大招商活动，取得丰硕成果。全年招商引资到位资金80亿元，与韩国希杰、香港华润、香港国资源、日本洋马等一批国际大公司、大财团的合作取得重大突破。把加大投入作为关键措施，集中抓了一批重大项目建设。全市固定资产投资完成200.9亿元，增长43.1%，创历史最高水平。聊城经济开发区建设取得重大进展。中华电力聊城发电厂2号机组、聊城热电机组扩建、华信铝业电解铝等重点项目已建成投产。济聊馆高速公路聊城东南连接线、聊阳一级路、临高路、临博路相继竣工通车，青银高速公路聊城段已开工建设。香江光彩大市场二期工程基本建成。水利、通信、电网改造等基础设施建设都取得了新的成绩。

（三）毫不放松地抓调整，经济结构进一步优化升级。按照“一产调优、二产调强、三产做大”的要求，狠抓了农村产业结构调整、“三个亮点”、百亿产业、县域经济和旅游业等项重点工作。三次产业比例达到22.1∶50.3∶27.6，二三次产业提高了3.5个百分点。农业结构继续优化，产业化水平提高，高效经济作物进一步发展，林牧渔业占农业总产值的比重达到32%。积极推进工业结构调整，速度效益同步提高，规模以上工业企业完成增加值增长26.7%，利税、利润分别增长45.9%和46%，有63户企业实现利税超千万元，其中11户企业过亿元。成功举办了第二届“江北水城文化旅游节暨国际龙舟节”，商贸流通业、旅游业、房地产业、金融保险业等都有了新的增长，社会消费品零售总额增长14.8%。营造“三个亮点”成效显著，实际利用外资1.22亿美元，比上年翻了一番，出口2.33亿美元，增长65.6%；高新技术产业产值增长47.1%，占规模以上工业总产值比重提高1.6个百分点；规模以上私营工业增加值、利润分别增长46.8%和72.8%，个体私营经济提供税收占地方财政收入比重达到29.2%。

（四）积极推进市场取向的改革，为经济社会快速发展增添了活力。坚持以改革促发展，深化国有资产管理体制改革，进一步完善了三个层次的国有资产监管运营体系。大力推进国有企业改革，城市信用社对外成功出让产权，东胶集团增资扩股进入实质性操作阶段。积极推进城市资产管理体制改革，公用事业领域改革有了良好开端。切实抓好城镇土地使用制度改革，实现政府土地收益6.4亿元。启动了31个强弱乡镇的改革发展试点。行政审批制度、住房制度、户籍管理制度、人事制度和财政体制等各项改革不断深化。下大力整顿规范市场经济秩序，“诚信聊城”建设开始起步。

（五）切实加强城市规划建设管理，城市面貌发生新的变化。江北水城建设取得新进展，正以新的形象走向全国、走向世界。全市城市建设开发投入达29亿元，其中聊城城区18.5亿元。聊城城市总体规划的修编工作已经完成。东北湖区清淤、古城区截污、徒骇河治污蓄水一期工程已全部完工，水城明珠、文化广场主体工程已经完工，谭庄水库、运河博物馆、古运河三期、人民广场等工程正在实施。城区道路、供排水、供气、供热等工程建设步伐加快。加大城区拆迁建设力度，完成拆迁面积100万平方米，竣工面积172万平方米。积极开展“五城”同创，我市被评为中国优秀旅游城市和省级园林城市。县市城区和小城镇建设取得了新的进展。

（六）更加重视经济社会协调发展，各项社会事业全面进步。大力实施科教兴聊战略，全年共取得重要科技成果98项；成功举办了“科技回访周”活动，引进了一大批科技成果。大力推进“以县为主”的基础教育体制综合改革，教育质量不断提高，高考录取人数比上年增加4911人，招飞工作夺得全国九连冠，职业教育、成人教育、民办教育进一步发展。高度重视卫生事业的改革与发展，建立了突发公共卫生事件应急预案，加强了疾病预防控制和传染病救治体系建设。积极推进文化体制改革，加强文化基础设施建设，丰富了群众文化生活。与聊城大学联合成功举办了山东省第十一届大学生运动会，全民健身活动和群众性体育活动广泛开展。完善计划生育工作新机制，保持了低生育水平，人口自然增长率为3.86‰。扎实开展土地市场清理整顿，确保了耕地面积的稳定。启动了生态市建设，加强重点污染源治理，环境质量逐步改善。积极开展严打整治斗争，认真化解各种社会矛盾，高度重视群众来信来访工作，有效维护了社会稳定。国防教育和双拥共建工作成效显著，我市被授予全国双拥模范城。新闻出版、广播影视、民族、宗教、外事、侨务、对台、人防、气象、地震、民政、残联、老龄、档案、史志和妇女儿童工作等都有新的发展。

（七）坚持执政为民，以高度负责的精神为群众办实事。加强就业和再就业工作，城镇新增就业再就业人员5.8万人，安置“4050”人员4310人，城镇登记失业率为3.2%。大力实施劳务输出战略，在外务工人员达到66.9万人，比上年增加14.2万人。高度重视社会保障体系建设，全市财政用于社会保障支出达7439万元，使“两个确保”、“一个低保”得到全面落实。认真帮助特困企业解决职工工资及补偿问题，市级财政总计支出2050万元，使企业职工生活得到较为妥善的安排。积极救助困难群众，全年共发放救助资金2617万元，使28.5万困难群众得到救助。积极推进农村税费改革试点，停征了农业特产税、乡村公益事业金，严格“两工”管理，暂停“一事一议”，对共同性生产费用严格压减，狠抓了涉农案件的查处，农民负担明显减轻。认真解决农民生产、生活实际困难，农村道路“村村通”工程投资4.2亿元，修建柏油路3480公里，使1801个村实现了通油路；投资5500万元，解决了300个村、26万人的吃水困难问题，提前一年完成了农村饮水三年攻坚计划；继续改造农村中小学危房，使办学条件得到进一步改善；在6个乡镇成功开展了新型合作医疗试点，受到农民欢迎。

（八）全面推进政府工作提速，经济发展环境明显改善。以建设“阳光政府”和“责任政府”为目标，扎扎实实地推进政府工作提速，深化行政审批制度改革，大力精减行政审批事项，提高办事效率和服务质量，市行政服务中心运行不断规范，各县（市、区）普遍建立了行政服务大厅。积极推行政务公开，建立了政府网站，设立了市长信箱，开通了市长热线。狠抓廉政建设，开展行风评议，加强审计监督，注重从源头上预防和治理腐败。深入开展“优化环境年”活动，发展环境得到进一步改善。

各位代表！过去一年我们遇到的困难比预料的大，取得的成绩比预料的好。成绩确实来之不易。这些成绩的取得，首先得益于党中央、国务院和省委、省政府对聊城工作的亲切关怀和大力支持，去年胡锦涛总书记和多位党和国家领导人来聊城视察，给全市人民以极大的鼓舞；得益于中共聊城市委的科学决策和坚强领导，特别是市委工作会议提出了“一二三四六”的总体工作思路，为今后的发展指明了方向，全市上下加快发展的热情空前高涨；得益于全市人民和社会各界的同心同德、团结奋斗，广大干部群众为实现率先崛起，积极进取，干事创业，表现出强烈的责任感和巨大的力量。在此，我代表市人民政府，向全市工人、农民、知识分子、各级干部，向各民主党派、工商联、无党派人士和社会团体，向驻聊人民解放军、武警部队官兵和广大公安干警，向中央和省驻聊单位，向所有关心、支持聊城发展的国内外朋友，表示衷心的感谢并致以崇高的敬意！

各位代表！过去一年取得的成绩令人鼓舞，但前进道路上仍然面临着不少矛盾、困难和问题。主要表现在：农民收入增长较缓慢，“三农”问题依然是经济社会发展中最薄弱的环节；经济结构性矛盾突出，第一产业所占比重过高，第三产业发展滞后，区域经济发展不够协调，国有企业改革和国有经济战略性调整步伐不快，外贸出口和利用外资规模小，经济的外向度较低；经济社会发展的体制性障碍仍然存在，有些领域的改革不到位，市场主体活力不强；社会事业改革发展相对滞后，社会保障能力不强，就业再就业压力较大，有些群众生产生活仍然比较困难；发展环境还存在不少问题，政府职能有待进一步转变，服务质量和工作效率不高的问题还比较突出；有的领导干部和国家工作人员廉洁自律较差，少数人甚至以权谋私，贪污腐败。我们要正视这些矛盾和问题，着眼长远，立足当前，抓住关键环节，采取有力措施，一个一个认真加以解决，尤其是对涉及群众切身利益的问题，更要千方百计调动力量，挖掘潜力，克服困难，认真及时地解决好。要牢记“两个务必”，时刻保持清醒头脑，倍加顾全大局，倍加珍视团结，倍加维护稳定，进一步增强压力感、紧迫感和责任感，认清形势，把握机遇，振奋精神，奋发有为，努力开创各项工作的新局面。

2004年政府工作的目标任务和工作重点

今年是全面建设小康社会、完善社会主义市场经济体制的重要一年，也是实现“十五”计划的关键一年。综合分析今年的形势，我市经济社会发展面临着良好的机遇和条件，国家宏观政策保持稳定，世界经济逐步回升，经济社会发展环境进一步改善。省、市委工作会议之后，全市已形成了解放思想、干事创业、加快发展的良好氛围，为做好今年的工作奠定了坚实的基础。特别是胡锦涛总书记来聊城视察，向我们提出了殷切希望和明确要求，极大地鼓舞了全市人民的士气，为我市加快发展带来了新的重大历史性机遇。可以坚信，只要我们抓住机遇，乘势而上，真抓实干，奋发进取，我们一定会取得新的成就，在全面建设小康社会的征程上迈出更大的步伐。

根据省委、省政府和市委的部署，今年工作的总体要求是：以邓小平理论、“三个代表”重要思想为指导，以胡锦涛总书记视察聊城为动力，全面贯彻落实党的十六大、十六届三中全会和中央、省、市经济工作会议精神，坚持市委工作会议确定的工作思路，坚持全面、协调、可持续的科学发展观，高度重视解决“三农”问题，以结构调整为主线，把招商引资、扩大投入作为长期的根本性措施，大力推进工业化和城市化，加快发展服务业，进一步推进各项改革，努力转变经济增长方式，不断优化发展环境，务求“三农”工作取得新成效，改革开放开创新局面，工业经济迈上新台阶，“三个亮点”实现新突破，城市面貌发生新变化，人民生活得到新改善，促进国民经济持续快速协调健康发展和社会全面进步。经济社会发展的主要预期目标为：国内生产总值增长15%，地方财政收入增长18%，新增城镇就业6万人，城镇登记失业率控制在4%以内，城镇居民人均可支配收入和农民人均纯收入分别增长8%和6.5%，居民消费价格总水平上涨2%。实际工作中要加倍努力，力争发展得更快更好。今年经济社会发展的工作重点是：

（一）把增加农民收入作为中心任务，努力解决“三农”问题。深入贯彻落实胡锦涛总书记来聊城视察的重要讲话精神，坚持把解决“三农”问题作为全局工作的重中之重，按照统筹城乡发展的要求，坚持“多予、少取、放活”的方针，促进农民增收、农业发展和农村经济社会全面进步。

进一步调整优化农业结构。实行最严格的耕地保护制度，加强基本农田保护，加快中低产田改造，抓好优质粮食基地建设，提高粮食综合生产能力，确保粮食安全。继续调整优化种植业结构，优化农业区域布局，大力发展蔬菜、林果、棉花、牧草、水产、花卉、药材、蚕桑等高效经济作物，扩大优质农产品比重。把发展畜牧业摆在农村经济发展的战略位置，努力在品种改良、规模饲养上实现新突破。加快林业产业化步伐，创造良好的经济效益和生态效益。

大力推进农业产业化。把培植龙头企业作为农业产业化的关键环节来抓，积极引导工商资本、民间资本和外资投向农产品加工业。落实扶持龙头企业的各项政策，努力培植壮大一批规模大、科技含量高、辐射带动力强的农产品加工运销龙头企业，全市重点抓好凤祥、蓝山等10家龙头企业的发展。积极发展农民新型合作经济组织，探索建立“龙头企业+各种经济组织+农户”的新型利益联结机制。大力推进农业标准化生产，有计划地发展一批无公害食品、绿色食品和有机食品生产基地。加强农产品质量标准体系、监测检验体系和动植物疫病防治体系建设，定期对农药、化肥、兽药进行检查，从源头上解决农产品药残超标问题。加强农产品市场建设，重点建设规模较大、档次较高的农产品交易批发市场，搞活农产品流通。

大力改善农村基础设施。建立多元化农业投入机制，加大对农业基础设施和农村社会事业的投入。提高财政支农比重，今年市级财政对农业的投入占财政支出的比重要达到7.4%，比去年增长16.5%。今年新增的教育、卫生等事业经费要主要用于农村。增强信贷支农力度，逐步建立政策性农业保险制度。加强水利基础设施建设，重点抓好谭庄水库、万家洼水库、胡家洼水库、一干渠衬砌、陶城铺灌区沉沙池清淤、位山灌区配套等工程建设，新打机井1万眼，发展节水灌溉面积 12.8万亩，新建乡镇供水工程54处。加强农产品流通设施建设，提高储存、保鲜能力和水平。

认真落实党在农村的各项政策。稳定农村土地承包关系，引导规范土地承包经营权合理流转。完善土地征用补偿办法，坚决防止和纠正随意侵占农民承包地、侵害农民利益的不法行为。继续深化农村税费改革试点和粮食流通体制改革，认真落实国家关于降低农业税税率的政策，放开粮食收购市场，把对粮食流通环节的间接补贴改为对农民直接补贴。加大治理“三乱”力度，加强农村“两工”管理，规范“一事一议”，加强对共同生产费用的监管，坚决防止农民负担反弹。

积极引导农村富余劳动力加快转移。进一步完善促进农村劳动力转移的投资政策、土地政策和户籍制度，建立城乡统一的劳动力市场，加强对农村劳动力的技能培训，搞好中介服务，促进农民向非农产业和小城镇转移。力争今年输出劳务新增10万人以上。依法保护进城务工农民的合法权益，下大力解决拖欠农民工工资问题。

（二）全力建设十大百亿产业，推动工业经济上规模、上水平。把工业化作为当前及今后一个时期结构调整的战略重点，坚定不移地实施工业带动战略，坚持走新型工业化道路，突出抓好十大百亿产业建设，推动工业经济上规模、上档次、上水平。

加快推进十大百亿产业发展。十大百亿产业是今后若干年工业经济发展的总抓手，要力争到2010年将有色金属、农业装备、造纸、食品、电力、汽车、纺织、化工、医药、电子信息等十大产业打造成为年销售收入达到或超过100亿元的大产业。增强百亿产业核心企业的责任感、使命感，瞄准世界先进水平，准确把握产业演进趋势和消费发展方向，找准发展定位，制定科学的发展规划和配套措施。鼓励与国际大公司、大财团进行产业对接，吸引跨国公司进行战略性投资，千方百计上大项目，不断壮大企业规模，提高核心竞争能力。以百亿产业核心企业为依托，积极吸引相关产业、产品加盟，引导中小企业搞好协作配套，拉长产业链，形成产业集群。抓好现有工业园区的整合和发展，鼓励大企业领办工业园区。继续实施名牌战略，争创更多的全省或全国名牌。

加大工业投入，培植新的经济增长点。坚持抓大投入、上大项目，全年工业投入力争突破175亿元。重点抓好聊城热电有限公司2×300MW机组扩建工程、鲁西化工集团工业园、高唐时风集团轻卡汽车、澳伸铝业电解铝及深加工、聊城华润30万纱锭项目、泉林纸业林浆项目、奥博特铜管三期、冠州集团20万吨镀锌板和彩涂板、凤祥乳制品产业化工程等238个在建和续建重点项目建设，力争尽快建成投产。抓好韩国希杰生物科技、中华电力聊城发电厂二期等重大项目的前期工作，力争及早开工建设。进一步加快市属工业发展，增强市本级工业经济实力。

切实转变工业经济增长方式。加快管理创新、机制创新和科技创新。用高新技术和先进适用技术改造传统产业，加快改造提升农用车、化肥、造纸、纺织、医药、机械配件等传统产业，进一步提高技术装备水平，培植一批名牌拳头产品。重点骨干企业要建立和完善技术研发中心，力争掌握核心技术，拥有自主知识产权。运用好两种资源、两个市场来发展工业，鼓励企业到外地或国外投资办厂。

（三）大力发展服务业，努力提高经济整体素质。从战略高度认识服务业，创新体制机制，优化发展环境，加大投入力度，不断提高服务业在国民经济中的比重。

高品位发展旅游业。坚持“政府主导、社会参与、市场运作”，发展大旅游，开发大市场，建设大产业，力争把我市建设成全省乃至全国的重要旅游热点城市。突出抓好大景区、大景点建设，尽快把江北水城风景区打造成为国内外著名的旅游风景区。深入挖掘聊城历史文化内涵，策划研究好古城区的保护和开发，建设独具特色、有影响带动力的旅游项目。加快古运河文化旅游线和水浒文化旅游线的开发建设。科学规划旅游线路，积极融入京津鲁大旅游圈。大力发展假日节庆经济，办好文化旅游节等活动。搞好旅游促销，大力开拓市场。加快建设一批具有聊城特色的餐饮、购物和娱乐场所，提高旅游业的市场竞争力。

积极发展商贸流通、现代物流和餐饮服务业。以建设区域性商贸中心城市为目标，科学规划，合理布局，努力构筑以新东方广场、聊建大厦、鲁西商厦、新开国贸城为核心的市中心商业区，以香江光彩大市场为核心的城区北部物流园区，以汽车产业园为核心的城区东部物流带，以新时代广场为核心的城区南部商业区。积极吸引国内外知名物流企业入驻聊城，构筑交通运输、仓储配送、信息网络等功能配套的现代物流体系。大力发展连锁经营，提高连锁经营在零售业中的比重。规划建设美食街、美食广场，提升聊城餐饮业的文化品位和风味特色。

加快发展交通、通信、房地产和新兴服务业。加强交通基础设施建设，重点抓好青银高速公路聊城段、蒙馆路聊城段、国道105线聊城段等重点工程建设，加快构建半小时经济圈。大力提高邮政、通信、广电的技术层次和服务能力，加快构筑高速、宽带、智能化信息传输平台。积极吸引外地资金参与我市房地产开发，重点发展面向中低收入阶层的经济实用住房。进一步放开搞活房地产二级市场。发展生产要素市场，鼓励兴办社区服务业，积极发展广告、工程、科技、会计、律师等服务业。

优化服务业政策环境。切实降低门槛，放宽市场准入，消除价格歧视，依法规范市场监管。创造良好的环境和条件，大力发展晚间经济。积极引导新的消费观念，扩大消费信贷规模，发展租赁消费，促进汽车、住房等新的消费热点形成。

（四）全力抓好招商引资，做大做强“三个亮点”。坚持把招商引资作为推动经济发展的总动力，大力培植外经外贸、高新技术、民营经济“三个亮点”，努力营造经济发展的新优势。

全力抓好招商引资。大力实施“大开放、大招商，促进大发展”战略，今年招商引资要达到100亿元，增长25%；实际利用外资2亿美元，增长60%以上，力争翻番。创新招商引资方式，采取小团组招商、蹲下来招商、专业项目招商、以商招商、网上招商等灵活多样的方式，进一步抓好对韩国、日本、港澳、台湾和江浙沪粤的招商，积极开展对欧美的招商。把大企业作为招商引资的重点，大力推动企业产权招商，力争在与世界五百强企业的合作上实现突破。拓宽利用外资的领域，积极引导外商投资城市建设、基础设施、第三产业、农业开发和加工贸易。加强专业招商队伍建设，尽快建立政府、社会、企业三个层次的招商体系。充分发挥聊城经济开发区招商主载体作用，举全市之力办好开发区，着力抓好韩国工业园、日本工业园的招商。进一步改善投资环境，重点解决好商务成本高、产业配套能力弱的突出问题，增强环境竞争力。

大力发展对外贸易。下大力推动和促进外贸出口，确保全年出口3.5亿美元，增长50%以上，力争增长80%。继续抓好纺织、机电、电解铝、纸制品、农副产品等优势产品出口，积极培育名牌出口产品。按照国际化、标准化的要求，发展一批优势农产品出口基地。大力发展加工贸易，重点办好经济园区的加工贸易区，搭建平台，搞好配套，吸引外商向加工贸易区聚集。积极组织企业参加各种大型经贸活动。鼓励有条件的企业和单位到境外投资办厂，承包工程和输出劳务。推进“大通关”工程，实行“口岸直通”运作模式，积极做好聊城一类口岸的争取工作。

加快发展高新技术产业。研究编制高新技术产业发展规划。围绕机电一体化、电子信息、新材料、生物技术、农业现代化等五大领域，依托东阿阿胶、阳谷电缆、冠洲集团、凤祥中科生物城等高新技术企业，培育一批科技含量高、开发潜力大、市场前景好的名牌拳头产品。以经济开发区为依托，以高新技术创业服务中心为支撑，加快规划建设市高新技术产业园。强化企业技术创新主体地位，加大政府科技投入。继续推进与高等院校和科研院所的合作，重点抓好北京、上海、西安、重庆、哈尔滨、南京、武汉等七个城市高校和院所科技成果的引进，为经济发展提供强有力的科技支撑。

放手发展民营经济。进一步确立民营经济与其他经济成分平等竞争、相互融合的市场主体地位，充分发挥民营经济在发展经济、繁荣市场、扩大就业、增加税收等方面的作用。放宽市场准入，着力优化发展环境，全面落实各项扶持政策，激发民营经济发展活力，形成农副产品加工、木材加工、铝制品、轴承、农机配件、建材等民营企业群体。建立健全以信用担保、诚信评价、法律服务、人才培训为主要内容的民营企业服务体系。培育壮大骨干企业，引导有条件的民营企业建立现代企业制度，提高经营管理水平，增强核心竞争能力。扩大民营企业自营进出口权，鼓励民营企业积极开拓国际市场。

（五）加强城市规划建设管理，促进区域经济协调发展。按照“三高”要求，规划建设和管理城市，更好地发挥城市对区域经济发展的辐射带动作用。

加强城市规划建设管理。坚持高起点规划，以建设卓越秀美的江北水城为目标，进一步完善聊城城市总体规划、专业规划和重大项目规划，重点抓好聊城市南区规划、县（市）城区和小城镇规划。坚持高标准建设，完成运河三期、东南湖区和东北湖区清淤改造等工程，尽快连通两河（运河、徒骇河）一湖（东昌湖），完善江北水城主体框架。抓好人民广场、文化广场、运河博物馆、湖南湖开发等工程建设。尽快启动“南展”计划，搞好“南区”起步区建设。抓好东昌路等12条道路的拓宽改造，扩大集中供热、供气的覆盖面，对6个城市出入口等重点地段进行高标准绿化。全面推行工程项目主体法人责任制，搞好建设项目的管理和市场化运作。坚持高效能管理，加快建立“政企分开、事企分开、监管分开、管养分工”的城市管理新体制。下大气力治污、治脏、治乱和治暗，全面开展“整治城区扬尘，建设清洁环境”活动，使城市的天更蓝、地更绿、水更清、夜更亮、城更净，努力争创全国卫生城市。积极推进城市经营，规范水务集团和江北水城集团的运作，加快园林、环卫、市政的市场化步伐，依法规范土地出让，实现土地收益5.6亿元，其中城区2.5亿元。加快临清、6个县城以及中心镇的建设，引导产业和人口向城镇集聚，城市化水平达到31%。

促进区域经济协调发展。研究聊城在全国、全省经济发展中的定位，加快接轨济南和半岛城市群，主动承接大城市的辐射。紧紧围绕强县、富民、富财政的总体目标，进一步深化改革，扩大开放，壮大工业，建设城镇，增强县域经济综合实力。切实扩大县域经济管理权限，营造县域经济加快发展的良好环境。鼓励条件较好的县市以全国、全省强县市为目标，奋力赶超，率先崛起。加大对欠发达县的扶持力度，支持这些县发挥优势，选准突破口，实现跨越式发展。全面落实对强乡镇和弱乡镇的激励扶持政策，深化乡镇经济体制和管理体制改革，优化发展环境，增强乡镇经济实力。

（六）加快推进市场取向的改革，在制度建设和体制创新上实现新突破。围绕完善市场经济体制，消除妨碍发展的体制性障碍，统筹兼顾，协调推进，重点在五个方面实现突破。一是深化国有资产管理体制改革。进一步完善责任、权利、义务相统一，管资产、管人、管事相结合的国有资产管理体制。继续探索有效的国有资产经营体制和方式，加快国有经济布局的战略性调整，确保国有资产保值增值。二是加快推进国有企业改革。建立归属清晰、权责明确、保护严格、流转顺畅的现代产权制度，通过发行股票、向外资和民营企业转让股权、实行员工持股等多种形式，推进投资主体多元化，发展混合所有制经济，使股份制成为公有制的主要实现形式。积极做好企业上市争取工作。继续推行年薪制、期权制、期股制。以产权制度改革为核心，加快中小企业彻底改制，实现国有资本退出。市属国有企业改革力争两年内基本完成。三是深化投融资体制改革。按照谁投资、谁决策、谁收益、谁承担风险的原则，确立企业的投资主体地位，凡符合国家产业政策和市场准入条件、企业自筹资金建设的项目，由企业自主决策，实行备案制。拓宽融资渠道，广泛利用外资，激活民间投资，建立投资稳定增长的内在机制。坚决防止盲目投资和低水平重复建设。四是深化社会事业改革。加快事业单位的综合配套改革，真正建立与市场经济体制相适应的管理体制和人事、分配制度。继续抓好城市公用事业改革，加快市场化进程。对有条件的医院、宾馆、招待所等事业单位积极稳妥地进行改制。积极推进文化、体育等社会事业的产业化、市场化。五是加快建设“诚信聊城”。建立健全信用监管体系和制度，整顿规范市场经济秩序，大力培育政府信用、企业信用和个人信用，塑造“诚信聊城”的良好形象。

（七）千方百计做好就业和社会保障工作，切实关心群众的生产生活。深入实施五大“民心工程”，诚心诚意地为群众办实事、解难事、做好事。一是努力扩大就业。力争全年新增就业人员6万人。全面落实扶持就业再就业的各项政策措施，扩大小额担保贷款覆盖面，支持下岗职工自主创业、自谋职业、灵活形式就业。加大再就业投入，着力开发公益性岗位，主要用于下岗职工再就业。大力实施就业困难群体帮扶工程，突出解决“4050”人员和一户两代、夫妻双方、抚养未成年子女的单亲家庭的再就业问题。加强职业教育和就业培训。加强劳动力市场信息网络建设，健全再就业援助制度。二是健全社会保障体系。继续巩固“两个确保”，完善企业养老保险市级调剂和财政备付金制度，加大扩面征缴力度，确保按时足额发放。认真做好低保工作，市级低保标准由去年的130元/月提升到今年的150元/月。各县（市、区）要根据财力情况，逐步提高低保标准。建立健全以基本医疗保险为主体的多层次医疗保险体系。稳妥推进企业离退休人员社会化管理服务，基本实现街道社区属地管理。三是实施“安康居住”工程。增加普通商品住房、经济适用住房和廉租住房供应，逐步解决中低收入家庭的住房困难。切实维护好城市改造、房屋拆迁过程中的群众利益。四是加快发展社区服务。建立完善城市社区管理和服务体系，积极开展面向社区居民的家政、养老、托幼、帮困、助残等项服务。五是下大力改善农民生产生活条件。积极发展农村卫生事业，重点建设改造好30所乡镇中心卫生院，加快推进农村新型合作医疗试点，建立对贫困农民的医疗救助制度。建立中小学辍学监控及贫困学生救助机制，确保不出现贫困生辍学；加快危房改造进度，用三年时间基本消除农村中小学危房，重点改造建设好乡镇中心小学和中心中学。加快实施农村道路“村村通”工程，新修公路4200公里，启动村村通客车工程。加大扶贫攻坚工作力度，使6.2万人口实现脱贫。

（八）积极做好财税金融工作，确保国民经济平稳运行。坚持增收节支并举，切实做好财税工作。进一步加大税收征管特别是主体税种的征管力度，严格依法治税、依率计征，做到应收尽收。进一步拓宽增收渠道，加强国有资产收益、土地收益、城市经营收益管理。按照建立公共财政的要求，把部分经营性事业单位退出财政供养范围。按照保工资、保稳定、保法定支出顺序，优先保证工资发放，优先安排好社会保障资金和救灾解困资金，除必保的政策性增支外，一般性支出一律实行零增长。加快预算编制改革，完善国库集中支付、政府采购、“收支两条线”制度。按照事权与财权相适应的要求，完善县乡财政体制。牢固树立过紧日子的思想，厉行节约，反对浪费，勤俭办一切事业。积极推进金融改革与发展。采取切实措施，深化农村信用社改革。建立相互依存、共同发展的良好银企关系，引导金融机构拓展金融产品，改进金融服务，增加信贷投放。深入开展“诚信金融”建设，强化全社会的信用意识，尽快把我市建成金融安全区。加快发展保险业，大力推进业务创新，提高服务水平。

（九）大力实施科教兴聊和人才强市战略，推动各项社会事业全面发展。坚持统筹经济社会发展，坚持把科技教育摆在优先发展的位置，坚持把人才资源作为第一资源，全面发展社会各项事业。

大力实施科教兴聊和人才强市战略。一是切实加强科技工作。进一步深化科技体制改革，充分调动科技人员的积极性。学习借鉴外地经验，向农村选派科技特派员，鼓励科技人员与农民共同创业。抓好5个重点农业科技园区建设，大力推广普及先进实用的农业新技术、新成果。加强企业技术研发中心建设，使企业真正成为技术创新的主体。大力发展各类科技中介服务组织，促进科技成果的推广应用。加强信息基础设施建设，推进国民经济和社会信息化。加强社会科学研究和成果的运用。二是加快发展教育事业。增加政府对教育的投入，进一步强化基础教育，巩固提高“普九”水平，加快发展高中阶段教育，努力提高教育质量。把农村教育作为重中之重，进一步完善农村义务教育管理体制，确保人事、经费管理到位；建立教师工资发放保障机制，确保按时足额发放。积极发展职业教育和成人教育，加快中等专业教育资源的优化配置，鼓励和支持社会力量以多种形式办学。大力支持高等教育发展，努力为聊城大学、聊城职业技术学院创造宽松的办学环境和条件。三是大力实施人才强市战略。树立人才资源是第一资源的理念，着力建设行政人才、企业经营管理人才和专业技术人才三支队伍，努力培养和造就大批适应现代化建设的各类人才。改革用人制度，创新工作机制，加强创业支持，努力创造有利于人才成长、创业、发展的良好环境。进一步完善政策，做好聊城籍大学生回聊安置工作，大力引进外地人才来聊城创业发展。

加快发展卫生、文化、体育等各项事业。大力推进卫生事业的改革与发展。切实加强公共卫生体系建设，用三年时间，建立健全市县两级疾病预防控制体系和突发公共卫生事件救治体系，提高公共卫生服务水平和应急能力。做好防治非典的各项准备，确保人民群众的身体健康和生命安全。加强医疗基础设施建设，扩建市人民医院，改善人民群众就医条件。深化文化体制改革，加强文化市场建设，重视发展社区文化和农村文化，大力发展文化产业。加强新闻出版、广播电视、网络等信息媒体的建设和管理。加强体育设施建设，重视竞技体育和体育产业发展，深入开展全民健身活动和群众性体育运动，努力提高全民健康水平。搞好国防动员、人民防空和交通战备工作，深入开展双拥共建活动，做好军队转业干部、退役士兵安置工作，支持驻聊部队建设，进一步提高双拥共建工作水平。大力做好民族、宗教、外事、侨务、对台、档案、史志、气象、防震、救灾、妇女儿童、老龄以及残疾人等项工作。

全面落实三大基本国策。全面推进生态市建设，努力争创国家环境保护模范城。着力抓好水污染和大气污染的防治，搞好水资源建设和小流域污染防治，加快污水处理厂建设，确保南水北调水质安全；坚持不懈地抓好企业污染治理工作，严格控制新上污染项目；重视农业和农村污染的防治。加快绿色聊城建设，大力植树造林，推进生态示范区和生态功能保护区建设。发展循环经济，突出抓好节水、节能、节材工作，积极开展资源综合利用。切实加强土地管理，严格执行国家关于土地管理的法规和政策，坚决制止乱占滥用耕地，加强土地复垦开发，促进集约利用，确保耕地总量动态平衡和耕地占补平衡。坚持不懈地抓好人口与计划生育工作，进一步完善工作机制，稳定低生育水平，提高人口素质。

（十）切实加强民主、法制与精神文明建设，努力保持社会稳定。各级政府要自觉接受人大及其常委会的监督，支持人民政协履行职责，做好人大代表建议、批评和意见的办理工作，加强与各民主党派、工商联、无党派人士的联系，积极支持工会、共青团、妇联等社会团体发挥作用。坚持民主集中制，推进决策科学化和民主化。完善基层群众自治性组织，保证人民群众依法行使民主权利。坚持依法治市，深入开展“四五”普法教育，提高全民的法律意识。大力开展爱国主义、集体主义、社会主义教育和崇尚科学、反对迷信的教育，倡导树立正确的世界观、人生观和价值观。认真贯彻实施《公民道德建设实施纲要》，提高全民的文明素质。大力开展“平安聊城”建设。牢固树立“安全第一”思想，认真落实“一岗双责”，强化基层基础工作，抓好专项整治，坚决杜绝各类重大恶性事故的发生。深入开展严打斗争，全面落实社会治安综合治理的各项措施，维护良好的社会秩序。高度重视和做好民族宗教工作。依法严厉打击“法轮功”邪教组织。认真做好人民来信来访工作，健全矛盾排查调处机制，及时处理群众反映的突出问题，努力预防和化解群体性、突发性事件，切实维护社会的安定团结。

进一步加强政府自身建设

完成今年的各项任务，各级政府必须按照为民、务实、清廉的要求，全面加强政府自身建设，努力提高决策水平和执政水平。

（一）要加强学习，努力提高执政能力和水平。各级政府工作人员特别是领导干部，要认真学习邓小平理论、党的十六大和十六届三中全会精神，深入实践“三个代表”重要思想，坚持立党为公、执政为民，努力学习市场经济、现代科技和金融知识，借鉴和吸收先进地区的经验，尊重群众的首创精神，努力提高五种能力。一是提高科学判断形势的能力。看形势要看本质、看主流，努力防止主观主义和片面性。善于用发展的办法解决新情况新问题，善于见微知著，防止苗头性问题演变为全局性问题。二是提高驾驭市场经济的能力。大力提倡解放思想、实事求是、与时俱进的精神，创新思路、创新体制、创新机制。三是提高应对复杂局面的能力。分析解决任何一个矛盾，都要考虑前因后果和相关因素，做到对上对下负责，对现实和长远负责，对局部和全局负责。四是提高总揽全局的能力。善于谋全局、管大事，自觉做到统筹城乡发展、统筹区域经济发展、统筹经济社会发展、统筹人与自然和谐发展、统筹国内发展和对外开放。五是提高解决实际问题的能力。树立正确的政绩观，把满足人民的需要、实现人民的愿望、维护人民的利益作为全部工作的根本出发点，使各项工作经得起实践、群众和历史的检验。

（二）要转变职能，加快建设法治政府。进一步完善重大决策的规则和程序，完善人民建议征集、重大事项社会公示、社会听证、专家咨询等制度，推进决策的科学化、民主化。全面实行政务公开，增强工作的透明度。进一步精减和规范行政审批，创新行政管理方式，推行政府部门行政效能评议，建立政府工作提速长效运行机制。推进电子政务建设，提高行政效率。《行政许可法》将于7月1日起正式施行，我们要积极适应《行政许可法》对政府工作的新要求，大力推进法治政府建设，努力提高依法行政水平。要将《行政许可法》纳入普法计划，采取多种形式宣传普及。要加强对政府公务员特别是行政执法人员的培训，切实规范政府工作人员的行为。凡是与《行政许可法》不一致的有关行政许可的规定，自7月1日起一律停止执行。要认真清理实施行政许可主体，凡未经公布的机关和组织，一律不得实施行政许可。要努力推进实施行政许可的制度创新，建立健全工作制度，完善工作规程。完善行政监督制度，坚持依法行政，切实做到有权必有责、用权受监督、侵权要赔偿。

（三）要改进作风，在狠抓落实上下功夫。各级政府及其工作人员要增强忧患意识，保持谦虚谨慎、不骄不躁的作风，保持艰苦奋斗的作风，时刻把党和人民的利益放在首位，真正做到权为民所用，情为民所系，利为民所谋，堂堂正正做人，清清白白为官，扎扎实实办事。市委、市政府确定今年为“工作落实年”，各级政府必须把狠抓落实作为转变工作作风的关键环节，进一步完善决策目标、执行责任、考核监督“三个体系”，分解任务，明确责任，一级抓一级，层层抓落实。大力弘扬求真务实精神，大兴求真务实之风，力戒官僚主义、形式主义，禁止搞任何形式的“政绩工程”、“形象工程”，坚决反对弄虚作假、浮夸虚报行为。下决心精减会议、文件和应酬活动，多到基层和群众中去，多深入到改革和建设第一线，多到困难大、问题多的地方，搞好调查研究，帮助基层和群众解决实际问题。对事关改革开放和经济发展的关键问题，对影响社会稳定的突出问题和群众反映强烈的热点、难点问题，要牢牢地记在心里、抓在手上，一件一件抓好落实，切实抓出成效，以实际工作取信于民。

（四）要清正廉洁，树立良好的人民公仆形象。进一步从源头上防治腐败，加强对政府工作人员特别是领导干部的反腐倡廉教育，强化廉政法制建设，认真落实廉政建设责任制、领导干部任期经济责任审计和述职述廉制度。严格执行中央、省和市委关于廉政建设的各项规定，做到廉洁自律，率先垂范。继续深入持久地开展反腐败斗争，把解决群众反映的突出问题作为反腐倡廉工作的重点，对侵犯公民权利、侵害群众利益的人和事，一经发现，要严肃查处；对执法犯法、徇私枉法、欺压百姓的腐败分子，要依法予以严惩，决不姑息迁就。坚持和发扬党的优良传统，勤俭办一切事业，反对讲排场、比阔气，反对铺张浪费，树立人民公仆的良好形象。

各位代表！当前，我市正处在加快发展的新阶段。展望未来，任重而道远，前景更美好。让我们紧密团结在以胡锦涛同志为总书记的党中央周围，以邓小平理论和“三个代表”重要思想为指导，在省委、省政府和市委的坚强领导下，解放思想，抢抓机遇，干事创业，加快发展，创造更加辉煌的业绩，为实现率先崛起和全面建设小康社会而努力奋斗！
